# The histone deacetylase inhibitor SAHA induces HSP60 nitration and its extracellular release by exosomal vesicles in human lung-derived carcinoma cells

**DOI:** 10.18632/oncotarget.6680

**Published:** 2015-12-19

**Authors:** Claudia Campanella, Antonella D'Anneo, Antonella Marino Gammazza, Celeste Caruso Bavisotto, Rosario Barone, Sonia Emanuele, Filippa Lo Cascio, Emanuele Mocciaro, Stefano Fais, Everly Conway De Macario, Alberto J.L. Macario, Francesco Cappello, Marianna Lauricella

**Affiliations:** ^1^ Department of Experimental Biomedicine and Clinical Neurosciences, Section of Human Anatomy “Emerico Luna”, University of Palermo, Palermo, Italy; ^2^ Euro-Mediterranean Institute of Science and Technology, Palermo, Italy; ^3^ Department of Biological, Chemical and Pharmaceutical Sciences and Technologies, Laboratory of Biochemistry, University of Palermo, Palermo, Italy; ^4^ Department of Experimental Biomedicine and Clinical Neurosciences, Laboratory of Biochemistry, University of Palermo, Palermo, Italy; ^5^ Department of Therapeutic Research and Medicine Evaluation, National Institute of Health (ISS), Rome, Italy; ^6^ Department of Microbiology and Immunology, School of Medicine, University of Maryland at Baltimore and IMET, Columbus Center, Baltimore, USA

**Keywords:** histone deacetylase inhibitor, SAHA, HSP60, oxidative stress, exosomes

## Abstract

HSP60 undergoes changes in quantity and distribution in some types of tumors suggesting a participation of the chaperonin in the mechanism of transformation and cancer progression. Suberoylanilide hydroxamic acid (SAHA), a member of a family of histone deacetylase inhibitors (HDACi), has anti-cancer potential but its interaction, if any, with HSP60 has not been elucidated. We investigated the effects of SAHA in a human lung-derived carcinoma cell line (H292). We analysed cell viability and cycle; oxidative stress markers; mitochondrial integrity; HSP60 protein and mRNA levels; and HSP60 post-translational modifications, and its secretion. We found that SAHA is cytotoxic for H292 cells, interrupting the cycle at the G2/M phase, which is followed by death; cytotoxicity is associated with oxidative stress, mitochondrial damage, and diminution of intracellular levels of HSP60; HSP60 undergoes a post-translational modification and becomes nitrated; and nitrated HSP60 is exported via exosomes. We propose that SAHA causes ROS overproduction and mitochondrial dysfunction, which leads to HSP60 nitration and release into the intercellular space and circulation to interact with the immune system. These successive steps might constitute the mechanism of the anti-tumor action of SAHA and provide a basis to design supplementary therapeutic strategies targeting HSP60, which would be more efficacious than the compound alone.

## INTRODUCTION

The International Agency for Research on Cancer has reported that 14.1 million new cancer cases and 8.2 million deaths due to cancer occurred in 2012 worldwide [1]. The report also indicates that the burden has shifted to less developed countries, which at the time accounted for 57% of all cancer cases and for 65% of deaths caused by cancer. Obviously, cancer incidence shows a tendency to increase as well as the number of deaths due to this disease, particularly in countries with low levels of economy, infrastructure and industry, which emphasizes once again the need to deal with this health problem globally, and the urgency of developing new effective anticancer therapies.

Possible and promising new targets for future anti-cancer strategies are the HSP-chaperones, which have been implicated in carcinogenesis for many years [2]. The roles of some of them, such as HSP70 and HSP90, have actually been investigated in some details and the molecular mechanisms involved have been elucidated to a considerable extent [3–11]. Much less is known about HSP60 (or HSPD1 according to the new nomenclature [12]). The hallmarks of cancer more generally accepted comprise six biological capabilities acquired during a multistep process. These capabilities include: sustaining proliferative signaling, evading growth suppressors, resisting cell death, enabling replicative immortality, inducing angiogenesis, and activating invasion and metastasis [13]. Recently, it has been indicated that tumour-derived exosomes can also be considered a hallmark of cancer [14]. In this regard, in previous work we showed that HSP60 reaches the plasma-cell membrane by a mechanism that involves lipid rafts and is released outside the cells in nanovesicles (exosomes) [15]. Furthermore, we and others have studied the quantitative variations of HSP60 during the development of various types of cancers and found that, in some of them, the levels increase progressively as the carcinogenic process advances [16–27]. We considered this quantitative change of interest, although it did not entirely support a direct role of HSP60 in cancer development since it could rather be a consequence of the disease. HSP60 also showed changes in distribution in cancer cells, appearing outside the mitochondria (its canonical place of residence) and being secreted outside the cell by an active mechanism [26–28]. Although HSP60 is not yet generally mentioned as a hallmark of cancer, these data strongly support that the degree of expression and levels of HSP60 are to be considered as hallmarks.

Here, to understand the mechanisms underlying the cascade of cellular events during carcinogenesis, we decided to investigate also potential post-translational modifications of HSP60. We tested the effect of suberoylanilide hydroxamic acid (SAHA), a compound known to have anti-tumor effects and to cause post-translational modifications of certain proteins. SAHA is a member of a family of histone deacetylase inhibitors (HDACi), which have anti-cancer effects [29–32]. This family of compounds is involved in the acetylation of histones and, thus, plays a critical role in chromatin remodeling and gene expression. HDACi also acetylate non-histone proteins and change their functions [33, 34]. Specifically, and pertinent to our work, HDACi compounds hyperacetylate the chaperones HSP70 and HSP90 altering their functions [35–38]. Use of SAHA (Vorinostat) was clinically validated in cancer patients resulting in its approval by the FDA in 2006 for the treatment of refractory cutaneous T-cell lymphoma [39]. More recently, several clinical trials of SAHA, either alone or in combination with other anti-cancer drugs (etoposide, gemcitabine, cisplatin, paclitaxel, doxorubicin, and cyclophosphamide), have been conducted in cancer patients and have shown antitumor activity in hematologic and solid tumors at doses that are well tolerated by patients [40–43]. Furthermore, a Phase I/II study showed that SAHA improves the efficacy of gefitinib (Iressa(®)) in EGFR-mutant non-small cell lung cancer [44].

The aim of this study was to investigate further how SAHA might interfere with cancer development and whether this interference might occur through an HSP60-mediated mechanism. We used the same tumor cell line with which we had previously studied the levels and distribution of HSP60 under various conditions pertinent to carcinogenesis, including its secretion via exosomes.

Here, we report on the cytotoxicity of SAHA in H292 lung-derived tumor cells and the mechanisms involved, focusing on HSP60.

## RESULTS

### SAHA cytotoxicity on H292 cells

**Figure 1:**
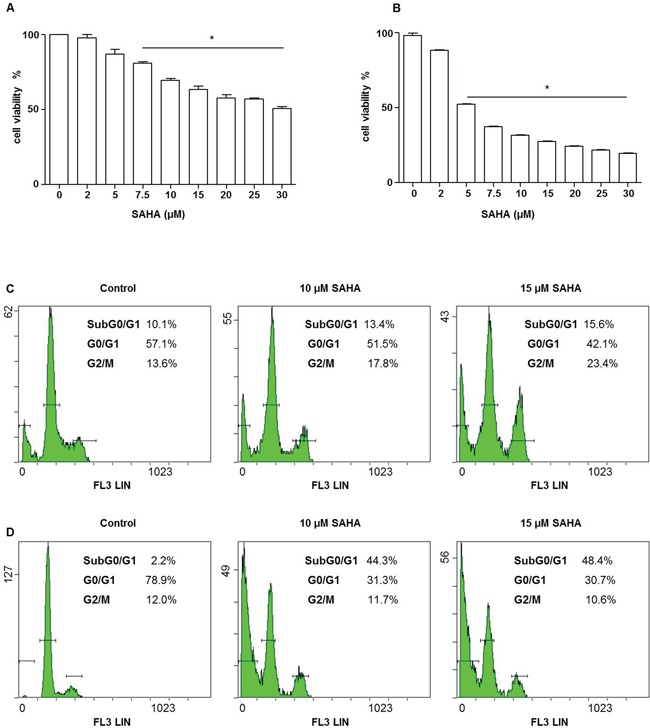
SAHA affects the viability and cycle of H292 cells Cell viability was assessed using the MTT test. A dose-dependent decrease of H292 cell viability was observed. The IC50 values for 24 **A** and 48 h **B** were 30 and 5 μM, respectively. Values are the means of three independent experiments ± SD (*different from control *p*<0.001). **C–D.** The graphs represent flow cytometry outputs showing cell cycle distribution, using propidium iodide DNA staining. Cells were treated with various doses of SAHA for 24 (C) and 48 (D) h. After 24 h (C) of SAHA treatment cells were predominantly arrested in the G2/M phase, whereas a pre G0/G1 peak appeared at 48 h (D), indicating DNA fragmentation.

To assess the cytotoxic effect of SAHA on H292 cells, we measured cell viability by the MTT assay. Cells were treated with increasing concentrations (within the range of 2-30 μM) of SAHA for various periods. The compound had a dose- and time-dependent cytotoxic effect, Figures 1A and 1B. After 24 h of treatment, SAHA reduced cell viability by 18% with 5 μM, by 40% with 15 μM, and by 48% with 30 μM (Figure 1A). The effect was even more pronounced at 48 h when 73% of cells were dead with 15 μM SAHA (Figure 1B). The doses causing 50% death (IC50) were estimated to be 30 μM and 5 μM at 24 and 48 h of SAHA treatment, respectively. Statistical analysis confirmed the results observed (Figure 1A and 1B, *p*<0.001).

### Effects of SAHA on the cell cycle

To investigate whether SAHA treatment has an effect on the H292 cell cycle, we performed flow cytometry after cell staining with propidium iodide (PI). We observed, using a range of SAHA doses, that at 24 h of treatment, the proportion of cells in G2/M phase was higher in cells treated with 10 and 15 μM (17.8 and 23%, respectively) than in the control untreated cells (13.6%) (Figure 1C). Prolonging the time of incubation with the compound to 48 h the amount of cells in G2/M phase decreased, while the percentage of cells in pre-G0/G1, characteristic of cells with fragmented DNA, increased reaching 44.3 and 48.4% with 10 μM and 15 μM of SAHA, respectively (Figure 1D).

### SAHA-induced ROS production and loss of mitochondrial membrane potential (Δψm)

**Figure 2:**
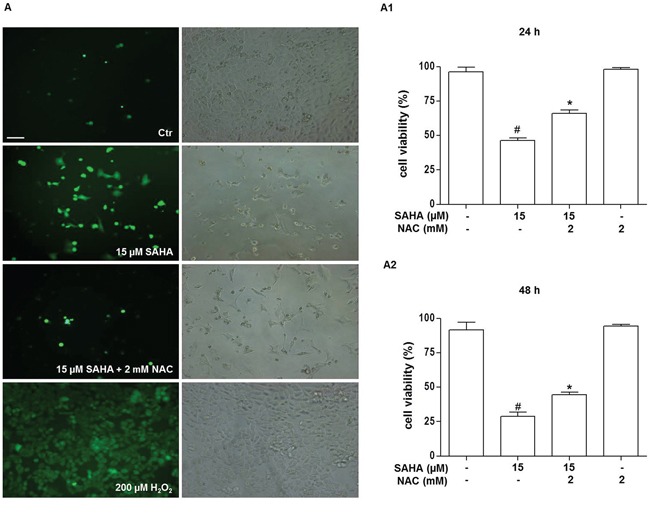
SAHA promotes ROS generation in H292 cells Cells were treated for 24 and 48 h with 15 μM SAHA in the presence or absence of 2 mM NAC. **A.** Representative photomicrograph of immunofluorescence showing ROS generation in H292 cells stained with H2DCFDA after 48 h of treatment with SAHA. (Bar=40 μm and magnification 200X for all images). A positive control for ROS generation was obtained by treating the cells for 10 min with 200 μM H_2_O_2_ (A, bottom right and left images). In (A) the left panels show H2DCFDA-stained cells visualized by FITC filter and the right panels the corresponding images without immunofluorescence. The effect of NAC on SAHA-treated H292 cell viability was evaluated after 24 (A1) and 48 (A2) h of treatment and measured by the MTT assay. In A, A1 and A2 the results are representative of four independent experiments. *p<0.001 vs control, 15 μM SAHA and 2 mM NAC ^#^*p*<0.001 vs control. Ctr = control.

Oxidative stress is one mechanism of cytotoxicity of HDACi on tumor cells [45–47]. Thus, to explore whether reactive oxygen species (ROS) are involved in SAHA-induced H292 cell death, the accumulation of intracellular oxygen radicals was assessed by fluorescence microscopy using the redox-sensitive fluoroprobe 5-(and-6)-carboxy-2′,7′-dichlorodihydrofluorescein diacetate (H2DCFDA), as described in Materials and Methods. We found a marked production of intracellular ROS after SAHA incubation compared to the control, untreated cells. This ROS production, which already appeared at 24 h of treatment with 15 μM SAHA (not shown), was even higher at 48 h (Figure 2A). The addition of the antioxidant N-acetylcysteine (NAC) counteracted the SAHA-mediated cell death as well as ROS production (Figure 2A, 2A1, and 2A2), suggesting that ROS production drives SAHA-induced cell death. As shown in Figure 2A1 and 2A2 there was a significant decrease of cells viability both at 24 h and 48 h of treatment with 15 μM SAHA alone and 15 μM SAHA in combination with 2 mM NAC compared to control (*p*<0.001). Moreover, we observed a significant increase of cell viability both at 24h and 48h of treatment with 15 μM SAHA and 2 mM NAC compared to 15 μM SAHA alone (*p*<0.001).

**Figure 3:**
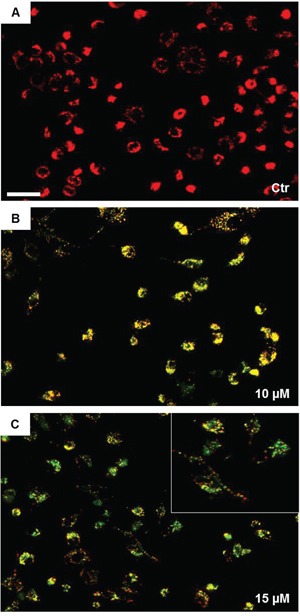
SAHA induces mitochondrial depolarization in H292 cells The mitochondrial membrane potential was evaluated by fluorescence microscopy using JC-1 staining. Images were acquired using a Leica DM5000 upright fluorescence microscope (Leica Microsystems) and merges were obtained using NIH Image J 1.40 program after acquisition. The mitochondrial depolarization was observed at 24 h with 10 μM SAHA and increased with 15 μM SAHA. **A, B** and **C** panels are representative images from three independent experiments. (Bar=100 μm for all images). The insert in (C) shows the details of depolarized cells. Ctr = control.

Since high levels of ROS are known to impair mitochondrial integrity, we next evaluated the effect of SAHA treatment on mitochondrial permeability transition (MPT), which represents an important parameter of mitochondrial function. The effect of SAHA on mitochondrial membrane potential (Δψm) was examined by staining H292 cells with JC-1, which specifically shows potential-dependent accumulation in depolarized mitochondria displaying a red to green fluorescence shift. The fluorescence data indicated that in control, untreated H292 cells red fluorescence was overwhelmingly predominant (Figure 3A), suggesting that most of cells were polarised. Exposure of cells to SAHA (10 and 15 μM) for 24 h caused Δψm depolarization, as revealed by the appearance and predominance of green fluorescence (Figure 3B and 3C). This indicated that mitochondria in most cells were depolarised as a consequence of SAHA treatment.

### SAHA lowered HSP60 levels

**Figure 4:**
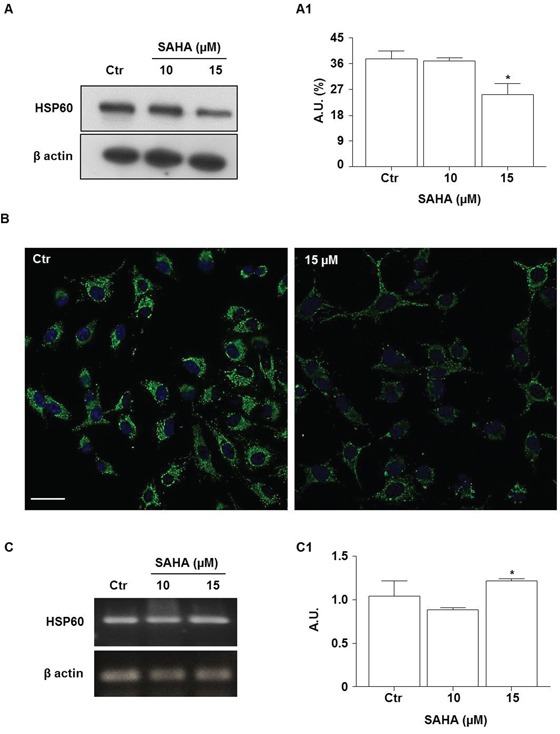
SAHA lowers the levels HSP60 protein **A.** Representative Western blotting of HSP60 levels after treatment with 10 and 15 μM SAHA for 24 h; (A1) Representative histogram of the densitometry analysis of protein bands. Bands intensities were normalized to those of the control in each series of experiments. (*p<0.01 vs control and 10 μM). A.U.= Arbitrary units. **B.** Representative images of immunofluorescence analysis of HSP60 cellular distribution in SAHA-treated cells. Nuclei were stained by Hoechst 33342, HSP60 was detected by a FITC conjugated antibody. Distribution of the chaperonin is shown by the merge of HSP60 and nuclei staining (Bar=30 μm for both images). **C.** RT-PCR analysis of mRNA HSP60 levels and (C1) densitometry of corresponding bands. HSP60 mRNA was significantly higher after treatment with 15 μM SAHA by comparison with 10 μM. Values are the means of three independent experiments ±SD (*p<0.01 vs 10 μM). For Western blotting and RT-PCR, β-actin was used as loading control. Ctr= control.

HSP60 is a chaperonin which is involved in the protection of cells from oxidative stress [48]. To determine whether SAHA treatment induces changes in HSP60 levels, we applied Western blotting analysis. As shown in Figure 4, the HSP60 levels were lower (1.5 fold) in H292 cells treated with 15 μM of SAHA for 24 h compared to the control. The reduction was significant compared with untreated cells and 10 μM SAHA (Figure 4A and 4A1; *p*<0.01). These data were confirmed by immunofluorescence (Figure 4B). In contrast, the measurements of HSP60 mRNA levels by RT-PCR revealed that SAHA treatment, in the doses of 15 μM, induced the up-regulation of HSP60 gene expression compared to 10 μM SAHA (Figure 4C and 4C1; *p*<0.01).

**Figure 5:**
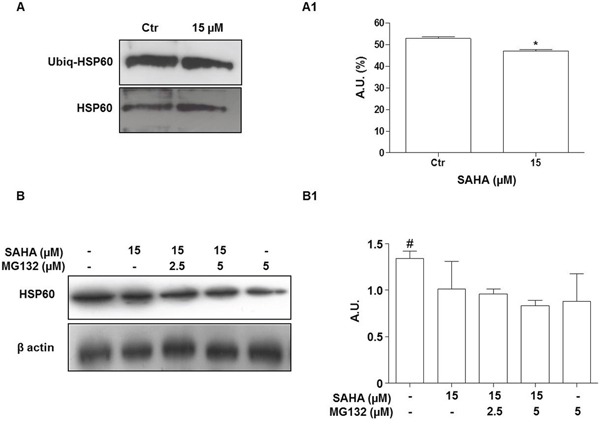
The proteasome inhibitor MG132 does not prevent the HSP60 decrease induced by SAHA **A.** Representative Western blotting of immunoprecipitation experiments after treatment with 15 μM SAHA for 24 h showing the HSP60 and ubiquitinated HSP60 (Ubiq-HSP60) bands. (A1) Densitometry of protein bands corresponding to the Ubiq-HSP60. The amount of ubiquitinated HSP60 was lower in treated cells than in the controls (*p=0.0464). A.U.=Arbitrary Unit. **B.** Representative Western blotting of HSP60 and (B1) densitometry of protein bands. H292 cells were treated for 24 h with 15 μM SAHA in the presence or absence of MG132. The correct protein loading was ascertained by immunoblotting for β-actin. MG132 did not prevent the HSP60 decrease induced by SAHA at any of the doses tested. The results are representative of three independent experiments. Values are the means of three independent experiments ±SD (^#^*p*<0.05 vs all other conditions). Ctr = control.

This first set of results suggested that the HSP60 levels were decreased depending on a post-transcriptional mechanism. In the following set of experiments we investigated whether proteasomal degradation could play a role in this process. An important step in the proteasomal degradation pathway is the formation of an ubiquitin-protein conjugate. Thus, we first investigated whether SAHA promotes HSP60 ubiquitination. After treatment of H292 cells with 15 μM SAHA for 24 h, cell lysates were subjected to immunoprecipitation with an anti-HSP60 antibody followed by immunoblotting with an antibody to detect ubiquitin-conjugated HSP60 (Figure 5A). The level of ubiquitinated HSP60 (Ubiq-HSP60) was lower after SAHA treatment compared to control untreated cells (Figure 5A1; p=0.0464). The addition of various doses of the proteasome inhibitor MG132 to SAHA-treated H292 cells did not prevent the drop of HSP60 induced by the HDACi at 24 h (Figures 5B and 5B1). HSP60 protein levels significantly decreased in all the conditions assayed compared to untreated cells (*p*<0.05). Collectively, these findings indicate that the involvement of the ubiquitin-proteasome system is unlikely in the diminution of HSP60 levels we observed in SAHA-treated cells.

### SAHA induced HSP60 post-translational modification: nitration

**Figure 6:**
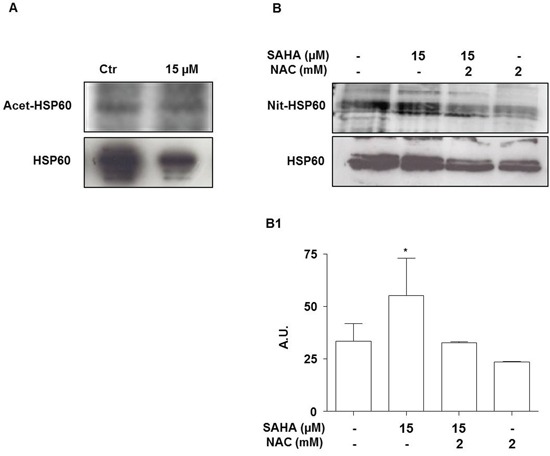
SAHA does not acetylate but promotes the nitration of HSP60 H292 cells were treated for 24 h with 15 μM SAHA alone or in combination with 2 mM NAC. **A.** Representative Western blotting of acetylated form of HSP60 (Acet-HSP60) and HSP60 corresponding bands. **B.** Representative Western blotting of nitrated form of HSP60 (Nit-HSP60) and HSP60 corresponding bands. (B1) Densitometry of the corresponding bands. The results are representative of three independent experiments. Values are the means of three independent experiments ±SD (*p<0.05 vs control and SAHA 15 μM with NAC 2 mM). Ctr = control; A.U.= Arbitrary Unit.

The previous observation that SAHA-treatment did not induce HSP60 ubiquitination, prompted us to investigate whether it might promote other post-translational changes. Recent investigations have demonstrated the potential of HDACi to acetylate chaperones such as HSP90 and HSP70 [49]. Thus, we investigated if SAHA would acetylate HSP60. Immunoprecipitation analysis demonstrated that in H292 cells the levels of acetylated HSP60 did not change after 24 h of exposure to SAHA (Figure 6A).

Next we evaluated the effect of SAHA treatment on other possible post-translational modifications. To this purpose we focused on nitration, a post-translational modification that is related to the production of reactive oxygen species [50]. HSP60 was immunoprecipitated with a specific antibody followed by Western blotting developed with anti-nitro-tyrosine antibody. A significant increase in the levels of nitrated HSP60 was observed after the incubation for 24 h with 15 μM SAHA compared to control and 15 μM SAHA in combination with 2 mM NAC (Figure 6B and 6B1) (*p*<0.05). Remarkably, the SAHA-induced increase of nitrated HSP60 was counteracted by the addition of 2 mM NAC, suggesting that nitration was associated with the oxidative events induced by SAHA. A three-dimensional model of HSP60 monomer showed that the tyrosines 222 (Y222) and 226 (Y226), at which nitration most likely occurred, are in the apical domain (Supplementary Figure S1). This domain is crucial for co-chaperonin and substrate binding by HSP60.

### SAHA increased secretion of HSP60 via exosomes

**Figure 7:**
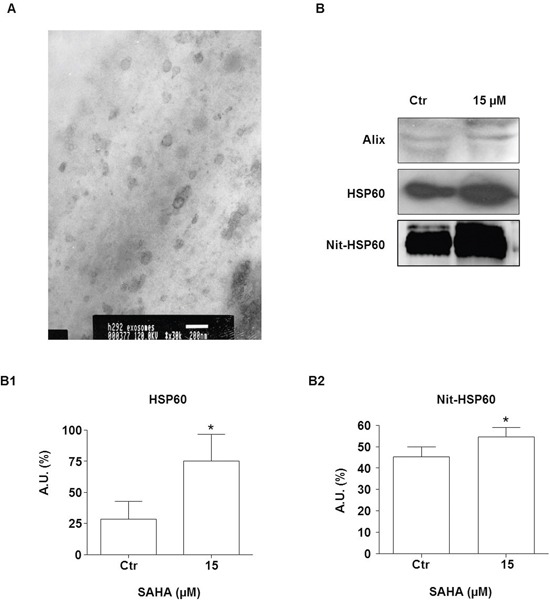
SAHA increases secretion of HSP60 via exosomes **A.** An illustrative image (TEM) for exosomes purified from H292 cells (bar=200 nm). **B.** Western blotting of exosomal Alix (upper panel B), HSP60 (middle panel B), and Nit-HSP60 (lower panel B) from the conditioned media of SAHA-treated and untreated (Ctr) H292 cells. Alix was used as exosomes marker. (B1) Densitometry of Western blotting for measuring HSP60 levels. (B2) Densitometry of Western blotting for measuring Nit-HSP60 levels in exosomes. The levels of both exosomal HSP60 and Nit-HSP60 were significantly higher in the exosomes from cells treated with 15 μM SAHA than in exosomes from untreated cells. The values are the means of three independent experiments ± SD (*p<0.01 vs control). Ctr = control; A.U.= Arbitrary Unit

Previous observations have shown that HSP60 is released in the extracellular microenvironment via the exosome pathway and this set of experiments was aimed at investigating whether SAHA effects on tumor cells involve inhibition of this pathway. First we analyzed our exosome preparations by TEM, and found evidence that the size and the morphology of the extracellular vesicles we obtained were those of typical exosomes [51] (Figure 7A). Western blotting showed that the exosome preparations expressed typical exosome markers, such as Alix [52] (Figure 7B, top row). HSP60 was detected in the exosomes from untreated cells and its levels were up to 2.7 fold higher after treatment with 15 μM SAHA (Figure 7B middle, and 7B1). In order to verify if exosomal HSP60 was nitrated, immunoprecipitation analysis was performed. The levels of nitrated HSP60 in the exosomes from cells treated with 15 μM SAHA for 24 h were significantly elevated by comparison with exosomes from untreated cells (Figure 7B bottom row and 7B2; *p*<0.01).

## DISCUSSION

The results of this study show that SAHA is cytotoxic for the human lung cancer-derived cell line H292 and that the cytotoxicity is associated with oxidative stress, mitochondrial damage, and decrease of intracellular HSP60 at the level of protein but not at the level of its mRNA. These effects are closely associated with a post-translational modification of HSP60, which becomes nitrated. Lastly, nitrated HSP60 is released outside the cell via exosomes.

The SAHA effect on H292 cells was dose- and time-dependent and was characterized by inhibition of the cell cycle at the G2/M phase, which was followed by accumulation of cells in subG0/G1 phase. These results are consistent with the findings by others showing that SAHA induces G2/M phase arrest by modulating the transcription of the cell cycle regulators [53], particularly down-regulation of Cyclin B1 or up-regulation of the CDK inhibitors p21 and p27 [54]. All these observations are also consistent with the ability of SAHA to block the cycle of tumour cells in G2/M phase.

The anti-tumor effects of SAHA and other HDACi drugs are associated with the accumulation of hyperacetylated histones, thereby causing modifications in chromatin structure and, consequently, affecting gene expression [29, 55]. Microarray analysis of the effects of HDACi drugs on gene expression has shown that these compounds modulate two sets of genes involved in pathways regulating cell cycle arrest or apoptosis [56, 57]. However, it has been argued that HDACi-induced chromatin remodelling cannot be the only mechanism responsible for the anti-tumor effect of these compounds [55]. Several non-histone proteins that are involved in cell growth, apoptosis, and differentiation can also be regulated by HDACi-mediated acetylation [46, 49].

Also, reversible acetylation regulates molecular chaperone functions [58]. Treatment with HDACi drugs caused hyperacetylation of HSP40, HSP70 and HSP90, affecting their functioning [59]. For example, HDACi drugs by inhibiting HDAC6 induce hyperacetylation of HSP90, leading to the dissociation of its client proteins ERB1, ERB2, and Akt [49].

To our knowledge, our data on the HDACi effects on HSP60 expression are new. We found that in SAHA-treated H292 cells the levels of HSP60 protein were significantly lower than in untreated cells, whereas the mRNA levels were slightly elevated. These divergent quantitative trends between HSP60 protein and mRNA indicate that the low levels of the HSP60 protein in SAHA-treated cells are most likely the result of post-transcriptional processes and do not involve gene expression down-regulation.

Thus, we turned our attention to possible post-translational modifications of HSP60 induced by SAHA that might lead to its degradation or other pathways that might result in its quantitative diminution inside the cell. In a previous work, we showed that geldanamycin induced hyperacetylation and decreased the level of mitochondrial HSP60 in osteosarcoma cells [60]. However, in this work we found that SAHA did not induce HSP60 acetylation in H292 cells.

Another alternative to explain the SAHA-associated low levels of HSP60 would call into play the ubiquitin-proteasome system (UPS), which would require ubiquitination of the chaperonin. However, our results do not support participation of the UPS. SAHA did not promote HSP60 ubiquitination in H292 cells and proteasome inhibition did not modify the effect of SAHA on HSP60 protein levels. However, a direct effect of the proteasome inhibitor on HSP60 protein level cannot be excluded, since it has been demonstrated that MG132 can block activation of NF-κB [61], a transcription factor for the chaperonin [62].

Thus, we investigated if a post-translational modification, other than acetylation, might occur in HSP60 in SAHA-treated cells. We focused on nitration and, indeed, found that in SAHA-treated cells nitrated HSP60 was present, most likely the modification occurring in the tyrosine residues, since we detected it by means of an anti-3-nitrotyrosine antibody. This result agrees with the observation that in rat insulinoma beta-cells nitration of HSP60 also occurred, triggered by chronic hyperglycemia [63].

The nitration of tyrosine residues to 3-nitrotyrosine is an oxidative post-translational modification [50]. Elevated levels of ROS in the presence of nitric oxide (NO) or NO-derived metabolites lead to the formation of reactive nitrogen species (RNS) such as peroxynitrite [64]. The RNS can modify tyrosine residues of many proteins resulting in protein nitration, which alters structure and functions of the target protein [65].

Our analysis using the fluorescent probe H2DCFDA provided evidence that, in H292 cells, SAHA increased the production of ROS, which were instrumental in the mechanism of cell death as suggested by the observation that the addition of the anti-oxidant NAC counteracted the drug's negative impact on cell viability. There is evidence supporting the idea that HDACi drugs lead to ROS production in various cancer-cell types [66, 36–37]. Furthermore, the induction of oxidative stress has been implicated in tumor-cell death induction by HDACi drugs [36, 67]. However, the underlying mechanisms by which HDACi drugs increase ROS production remain poorly defined. It has been suggested that it may depend on the inhibition of thioredoxin [68], a ubiquitous protein which operates as an intracellular scavenger of ROS [67]. For example, it has been reported that HDACi drugs increase the expression of thioredoxin-binding protein (TBP-2), a molecule that inhibits thioredoxin through a direct binding with this protein [69]. This inhibition causes imbalance between oxidants and antioxidants, in turn leading to increased ROS levels.

It has also been reported that HDACi drugs increase the production of ROS as a result of mitochondrial injury. In this regard, it has been reported that Trichostatin A weakened the activity of the mitochondrial respiratory chain by decreasing the expression of some subunits of complexes I and III, which lead to generation of ROS [36]. Thus, ROS increase leads to disruption of the mitochondrial membrane potential and cell death. In line with this observation, we demonstrated that SAHA induced in H292 cells dissipation of the mitochondrial membrane potential, possibly as a consequence of ROS increase. Thus, we can postulate that there is a link between ROS increase and nitration of HSP60. This idea is supported by our finding that the addition of the anti-oxidant NAC prevented the nitration of HSP60 induced by SAHA.

Tyrosine nitration modifies key properties of amino acids [50]. Likewise, the incorporation of a nitro group to a tyrosine can lead to structural and functional changes in a protein, which may alter cell homeostasis [70]. Protein tyrosine nitration has been detected in a variety of pathological conditions such as cardiovascular disorders, diabetes, and hepatic and neurodegenerative diseases [71, 72]. The excess of ROS and RNS in the mitochondria can lead to the formation of peroxynitrite and thereby increase tyrosine nitration in mitochondrial proteins. Nitration of mitochondrial proteins can further deteriorate the organelle's functioning and thus interfere with cell maintenance and survival [73]. This is illustrated by the demonstration that nitration of a single tyrosine residue in HSP90 is sufficient to convert HSP90 from a pro-survival protein into a mediator of motor neuron death [74]. Nitration of the HSP90 at position 33 induces conformational changes and its translocation to the mitochondrial outer membrane, in which it probably interacts with one or more mitochondrial proteins and thereby altering mitochondrial activity, a sequence of events that may be associated with pro-survival mechanisms in cancer cells [75].

It has been shown that nitration of HSP60 not only causes a significant decrease in its ATP-hydrolysis activity but also it disturbs the interaction of HSP60 with its co-chaperonin HSP10 [63]. The sites for co-chaperonin and substrate binding are both localized in the apical domain of HSP60 [76]. The three-dimensional model of HSP60 monomer we constructed (Supplementary Figure S1) shows that in the apical domain are present the highly conserved Tyr222 and Tyr226 [63, 76]. Nitration of one or both these tyrosine residues in the apical domain of HSP60 could abolish substrate binding and interfere with its folding. In turn, protein misfolding, or lack of folding, due to HSP60/HSP10 dysfunction surely will alter mitochondrial functionality. Thus, in our experimental system, HSP60 nitration could negatively affect its chaperoning function and, thereby, generate the mitochondrial dysfunction we observed in SAHA-treated H292 cells.

At this point, it was unclear why intracellular HSP60 would be low in SAHA-treated cells: our data showed that HSP60 gene expression was not down-regulated, and that the UPS was not involved either. Therefore, in order to explore potential mechanisms underlying the decrease of the HSP60 intracellular levels in SAHA-treated cells, we looked for possible involvement of exosome-mediated extracellular release. Exosomes are cell-derived vesicles that are released by many cell types and are also present in body fluids [77]. These vesicles have been proposed as key elements of intercellular communication in normal and pathological conditions, including tumors [51, 78], in which they may have a role in chemoresistance [79].

We have already shown that HSP60 is secreted by H292 tumor cells but not by normal cells, and that the secretion involves lipid rafts and exosomes [26, 27]. Moreover, we showed that HSP60 is secreted by exosomes from colon cancer tumor cells both *in vitro* and *in vivo* and that the levels of HSP60-carrying exosome in plasma decrease after surgical excision of the tumor [80]. The current results show that SAHA treatment causes the release of exosomes containing nitrated HSP60. The SAHA-induced exosome production, occurring along with ROS overproduction, could result from ROS stimulation. In agreement with this hypothesis, it has been reported that ethanol-induced ROS lead to an increase in the production of exosomes by cardiac myocytes [81].

Many studies have been focused on extracellular HSPs and their biological significance outside cells [15, 51, 82]. For instance, tumor-derived exosomes have been used as a source of tumor antigens to induce anti-tumour immune responses [82]. Exosome-bound HSPs recognize receptors on immune system cells [83]. The amount of secreted exosomal HSP60 significantly increased after treating HepG2 cells with irinotecan hydrochloride and carboplatin [5]. The HSP60-carrying exosomes derived from HepG2 cells treated with irinotecan hydrochloride and carboplatin elicited a strong cellular immune anti-tumour response [5]. In this regard, it is pertinent to note that HDACi drugs are capable of enhancing the immunogenicity of cancer cells. Several groups have reported the upregulation of natural killer (NK)-cell activating ligands, MHC class I and II molecules, and components of the machinery for antigen presentation, and the increase of co-stimulatory molecules on the surface of cancer cells resulting from exposure to HDACi drugs [83]. In addition, it has been reported that the HDACi MS-275 caused overproduction of exosomes in HepG2 cells [84]. This was paralleled by high levels of immuno-stimulating proteins, e.g, HSP70, in the exosomes.

We provide evidence for the first time that exosomes released from H292 cells contain a nitrated form of HSP60 and that this form increased after treatment with SAHA. What would be the role, if any, of nitrated HSP60 in immune responses? On this issue, some information, controversial however, is available indicating that protein nitration is linked to tumor cell evasion from T lymphocyte-mediated immune response [85]. It has also been reported that nitration of EGF and TNFα makes these molecules strongly immunogenic [86], and that the presence of nitrotyrosine-modified proteins is associated with several autoimmune diseases [87]. In conclusion, HSP60 is a chaperonin with an emerging role in carcinogenesis. Its levels are increased in a number of neoplasms in which it may be found intra- and peri-cellularly and in circulation, and high intracellular levels accompany uncontrolled proliferation and neoplastic transformation [17–27, 88, 89]. In the present study, we provide evidence for the ability of SAHA to modify levels and biochemical characteristics of HSP60, and induce its secretion via exosomes in a tumor-cell line. SAHA is an HDACi drug that causes anti-neoplastic and pro-apoptotic effects in a variety of tumour systems with low toxicity toward normal cells [29, 29–32]. The molecular mechanisms, in which nitrated HSP60 is involved in tumor-cell death, and the action of exosomes carrying the modified chaperonin when they arrive at their destination (possibly cells of the immune system) in organisms treated with SAHA, remain to be elucidated. However, the data already available encourage creative thoughts to design anti-cancer therapeutic strategies, using SAHA together with manipulation of the molecules it influences, such as HSP60.

## MATERIALS AND METHODS

### Antibodies

Anti-HSP60 (clone LK1) monoclonal antibody was from Sigma (Sigma-Aldrich, St. Louis, MO) and used diluted 1:1,000; anti-Alix monoclonal antibody was from Pharmingen (BD Biosciences, San Diego, CA) and used diluted 1:500; rabbit polyclonal antibodies against acetylated lysine was from Cell Signaling Technology (Cell Signaling Technology, Danvers, MA) and used diluted 1:1,000; mouse monoclonal antibody against 3-nitrotyrosine was from Abcam (Abcam, San Francisco, CA) and used diluted 1:1,400; and mouse monoclonal antibody against ubiquitin (clone P4D1) was from Santa Cruz Biotechnology (Santa Cruz, CA) and used diluted 1:1,000; anti-Actin HRP (Clone C-2) monoclonal antibody was from Santa Cruz Biotechnology and used diluted 1:5,000; and horseradish peroxidase (HRP)-conjugated sheep anti-mouse antibody and Protein-G/A Sepharose were from Santa Cruz Biotechnology.

### Cell culture and reagents

Mucoepidermoid tumor H292 cells were obtained from the American Type Culture Collection and cultured as monolayer in RPMI 1640 medium supplemented with 10% foetal calf serum (FCS), 2 mM glutamine, 50 U/ml penicillin and 50 μg/ml streptomycin at 37°C in a humidified atmosphere containing 5% CO2. Before each experiment, except where noted differently, cells were seeded in 96 or 6-well plates and were allowed to adhere and grow until 70% confluency when were treated with SAHA (Sigma-Aldrich, Milan, Italy), N-acetylcysteine (NAC, Sigma-Aldrich), MG132 (Calbiochem, Darmstadt, Germany), or vehicle only, for 24 or 48 h. Stock solutions of SAHA or MG132 were prepared in dimethylsulfoxide (DMSO, Sigma-Aldrich) and diluted to the tested final concentrations in the culture medium. The final concentration of DMSO did not exceed 0.04%.

### Cell viability determinations

To determine cell viability after SAHA treatments (0-30 μM), the MTT (3-(4,5-dimethylthiazol-2-yl-2,5-diphenyl tetrazolium bromide) assay (Sigma-Aldrich) was performed as previously reported [90]. Briefly, 20 μl of fresh MTT solution (11 mg/ml) were added to the cells and incubation was prolonged for 2 h at 37°C and 5% CO_2_. The reaction was stopped replacing the medium with 100 μl of lysis buffer (20% SDS, 10% dimethylformamide and 20% acetic acid). After solubilisation, absorbance was read at 570 and 690 nm. Each condition was tested six times and results were analysed for statistical significance. Based on MTT results, the SAHA concentrations chosen for all experiments were 10 and 15 μM.

### Cell cycle analysis

Treated and control untreated cells were trypsinized, harvested, and centrifuged 120 x g for 10 min. The analysis of cell cycle distribution was performed as previously described [91]. Briefly, cell pellets were suspended in 1 ml of phosphate-buffered saline (PBS) and were fixed in 70% ice-cold ethanol (v/v) for 1 h at 4°C. Then cells were centrifuged at 120 x g for 10 min and suspended in a hypotonic fluorochrome solution (50 μg/ml propidium iodide, 0.1% sodium citrate, 0.1% Nonidet P-40 and 100 μg/ml RNase A) and incubated overnight in the dark at 4°C prior to flow cytometry analysis. DNA content was measured in a Beckman Coulter Epics XL flow cytometer (Beckman Coulter Inc, Brea, CA) and data were analysed using Expo32 software.

### Analysis of mitochondrial membrane potential

The mitochondrial transmembrane potential in H292 cells was measured using the cationic carbocyanine dye JC-1 assay (Cayman Chemical Company, Ann Arbor, MI) as previously reported [92]. JC-1 is a lipophilic cationic dye that forms aggregates resulting in a red emission (emission of JC-1 aggregated shows at 590 nm) in normal polarized mitochondria, but it forms monomers that emit green fluorescence (emission of JC-1 monomeric shows at 525 nm) when mitochondrial membrane potential collapses. As a consequence, the loss of mitochondrial membrane potential is indicated by a decrease in the red-to-green ratio [90]. After treatment with SAHA, cells (8×10^3^/well) were incubated for 15 min at 37°C in medium containing JC-1. Then, cells were washed twice with PBS and examined using a Leica DM5000 upright fluorescence microscope (Leica Microsystems, Heidelberg, Germany).

### Fluorescence detection of intracellular oxygen radicals

Generation of reactive oxygen species (ROS) was estimated by 5-(and-6)-carboxy-2′,7′-dichlorodihydrofluorescein diacetate (H2DCFDA, Molecular Probe, Life Technologies, Eugene, OR), a probe that, upon oxidation by ROS, yields a fluorescent adduct that is trapped inside the cell producing a diffuse green fluorescence. For these experiments, cells (8×10^3^/well) were treated with compounds for various times, then medium was removed and cells were incubated with 100 μM H2DCFDA for 30 min in Hank's balanced salt solution (HBSS). Fluorescence analyses were performed in a Leica DMR microscope equipped with a DC300F camera (Leica Microsystems, Wetzlar, Germany) with a FITC filter (excitation wavelength of 485 nm and emission wavelength of 530 nm). Representative pictures were selected and acquired by Leica Q-Fluoro Software.

### Protein extraction and quantification

Treated (for example with SAHA and/or other compounds as indicated when pertinent) and untreated cells as controls were lysed into ice-cold lysis solution containing radioimmunoprecipitation assay (RIPA) buffer, as previously described [27]. Lysates were then spun at 16,000 x g for 30 min at 4°C. The supernatant was recovered, the protein concentration determined by Bradford assay using albumin bovine serum (BSA, Sigma Aldrich) as standard and then stored at −80°C until use. This preparation is referred to as “total-cell lysate.”

### Purification of exosomes from conditioned medium

Exosomes were isolated as previously described [27, 28]. Eighty ml of conditioned medium from 80×10^6^ H292 cells were collected after 24 h of SAHA treatment in serum-free medium, and centrifuged (800 × g for 10 min) at 4°C to eliminate cells and debris. The cell- and debris-free medium was collected on ice and centrifuged at 13,000 × g for 20 min at 4°C to bring down and eliminate small cellular debris and mitochondria. The supernatant was collected and exosomes were separated by centrifugation at 110,000 × g for 2 h at 4°C using a Sorvall WX100 Ultra Series ultracentrifuge (Thermo Scientific, Milan, Italy). The exosome pellet was collected, washed once in PBS and then suspended in 100 μl of PBS containing proteases inhibitors. Proteins in exosomal preparations were quantified with the Quant-iT™ protein assay kit (Invitrogen Molecular Probes, Italy), using the Qubit fluorometer according to the manufacturer's instructions (the kit is accurate for protein concentrations ranging from 12.5 μg/ml to 5 mg/ml).

### Exosomes validation by transmission electron microscopy (TEM) and alix detection

Exosome pellets purified from the cells were firstly examined by TEM to ascertain the presence of exosomal vesicles, whose diameter has to be 100 nm or less [27, 28]. Pellets were suspended in residual fluid from PBS wash, followed by addition of 100 μl freshly made fixative (2.5% glutaraldehyde in PBS) to preserve vesicle structure and morphology. Preparations were mounted on formvar nickel 300-mesh grids by layering grids over 10 ml drops of exosome preparations for 10 min at 24°C. Grid-mounted preparations were stained with uranyl acetate and lead citrate, and subsequently examined with a JEOL JEM 1220 TEM at 120 kV. Also, the presence of exosomes was confirmed by Alix detection using Western Blotting as described below.

### Western blotting

Western blotting was performed as previously described [93]. Briefly, 40 μg of proteins from total cell lysates or from exosome preparations was added to 4X Laemmli buffer and heated for 5 min at 95°C. Proteins were resolved by 12% SDS-PAGE along with a molecular weight marker (Bio-RAD laboratories, Milan, Italy). Proteins were then transferred to nitrocellulose membranes. The membranes were stained with Ponceau S to verify the quality of transfer and loading similarity. Before applying antibodies, the membranes were blocked with 5% BSA, and probed for 12 h with the specific antibody, followed by incubation with horseradish peroxidase-conjugated secondary antibody if necessary. The final detection was performed using ECL Western Blotting Detection Reagent (Amersham Biosciences, GE Healthcare Life Science, Milan, Italy), according to the manufacturer's instructions. Densitometric analysis of the bands was performed using the NIH Image J 1.40 analysis program (National Institutes of Health, Bethesda, MD).

### Immunoprecipitation analysis

In order to detect protein post-translational modifications, immunoprecipitation was performed as previously described [60]. Briefly, 5 μg of anti-HSP60 antibody per 1ml of total cell lysate or per 1ml of exosomes pellet, were incubated overnight at 4°C with gentle rotation. Antibody/protein complexes were then immunoprecipitated with antibodies linked to Protein-G/A Sepharose beads. Nonspecifically bound proteins were removed by repeated washings with isotonic lysis buffer. Immunoprecipitated proteins were resolved by 12% SDS-PAGE using primary antibodies against acetyl lysine, or against 3-nitrotyrosine, or against ubiquitin. Each experiment was performed three times.

### Bioinformatics: 3D model of HSP60

In order to show the position of possible nitration sites in human HSP60, a three-dimensional model of the protein was done as previously described [94, 95]. The sequence was retrieved from the PubMed website (http://www.ncbi.nlm.nih.gov/genbank/), using the accession number NM_002156. The three-dimensional model of HSP60 monomer was build using a fully automated protein structure homology-modelling server named SWISS-MODEL (http://swissmodel.expasy.org/) accessible via the ExPASy web server (http://www.expasy.org/). The model was visualized and modified by PyMol (http://www.pymol.org).

### RNA extraction and RT-PCR analysis

Total cellular RNA from untreated and treated cells was extracted with TRI REAGENT® (Catalog Number T9424, Sigma-Aldrich) according to the manufacturer's instructions. RT-PCR was conducted as previously described [96]. cDNA was synthesized using ImProm–II Reverse Transcriptase (Catalog Number A3800, Promega Corporation, Madison, WI). To obtain 200 ng of cDNA, 20 μl of mixed solution with RNA template, 0.5μg Oligo (dT) primers, 6 mM MgCl2, 5X reaction buffer, 1 μl dNTP (0.5 mM mixture of dATP, dCTP, dGTP, and dTTP), 20U RNase inhibitor, 1 μl ImProm-II reverse transcriptase, and nuclease-free distilled water were incubated at 25°C for 5 min and then at 42°C for 90 min. To inactivate reverse-transcriptase, the mixture was incubated at 70°C for 15 min. Thereafter, cDNA was amplified using GoTaq® Flexi DNA Polymerase (Catalog Number M8291, Promega Corporation, Madison, WI), using 1.25U GoTaq Polymerase. Semi-quantitative PCR was performed by adding specific primers for human HSP60 and β-actin which was used as housekeeping gene internal control (Table 1). Reactions were carried out with 2 min of preheating at 95°C and 35 repeated cycles of reactions at 95°C for 1 min, 60°C for 1 min, and 72°C for 3 min. A final incubation was done at 72°C for 5 min, and the reaction was stopped at 4°C. The PCR product was visualized on 1.5% agarose gel with the SYBR stain (SYBR Safe TM DNA gel stain, 10,000X concentrated in DMSO- Invitrogen, Carlsbad, CA). Experiments were performed in triplicate. Quantitative measurements of bands were performed using the NIH Image J 1.40 analysis program (National Institutes of Health).

**Table 1:** Forward and reverse primers used for the polymerase chain reaction

Species	Primer	Forward	Reverse
*Homo sapiens*	HSP60 var1	5′-GAG TAG AGG CGG AGG GAG-3′	5′-AGT GAG ATG AGG AGC CAG TA-3′
	H-ACTIN β	5′-CAC CTT CAC CGT TCC AGT TT-3′	5′-AGG TAC TCC GTG TGG ATC GG-3′

### Immunofluorescence

Immunofluorescence was performed as previously described [97]. Cells were fixed with ice cold methanol for 30 min, washed in PBS, pH 7.4, and then were incubated with the unmasking solution (10 mM trisodium citrate, 0.05% Tween 20) for 10 min at 23°C. Then, the cells were incubated with the blocking solution (3% bovine serum albumin in PBS) for 30 min at 23°C and with the pertinent anti-HSP60 primary antibody diluted 1:500, overnight at 4°C. The next day, the cells were incubated with FITC conjugated secondary antibody diluted 1:250 for 1 h at 23°C. The nuclei were counterstained with Hoechst 33342 (Sigma-Aldrich) for 15 min at 23°C. Finally, all slides were mounted with cover slips using a drop of Vectashield (Vector, Burlingame, CA). The slides were observed and images were captured using a confocal Leica DMI 6000B microscope (Leica Microsystems, Heidelberg, Germany).

### Statistical analyses

Statistical analyses were performed using statistical software package GraphPad PrismTM 4.0 software (GraphPad PrismTM Software Inc, San Diego, CA). Comparisons were carried out between the control (untreated) vs all by unpaired *t*-test and One-way analysis of variance. If a significant difference was detected by ANOVA analyses, this was further evaluated by Bonferroni post-hoc test. The data were expressed as means ± SD. The statistical significance threshold was established at the level of p≤0.05.

## SUPPLEMENTARY FIGURE


