# Supplementary material for: The histone deacetylase inhibitor SAHA induces HSP60 nitration and its extracellular release by exosomal vesicles in human lung-derived carcinoma cells
Source: Oncotarget. 2015 Dec 19;7(20):28849–67. doi: 10.18632/oncotarget.6680 (PMC5045361; doi:10.18632/oncotarget.6680)
Supplement: Supplementary file 1 [file oncotarget-07-28849-s001.pdf]

SUPPLEMENTARY FIGURE

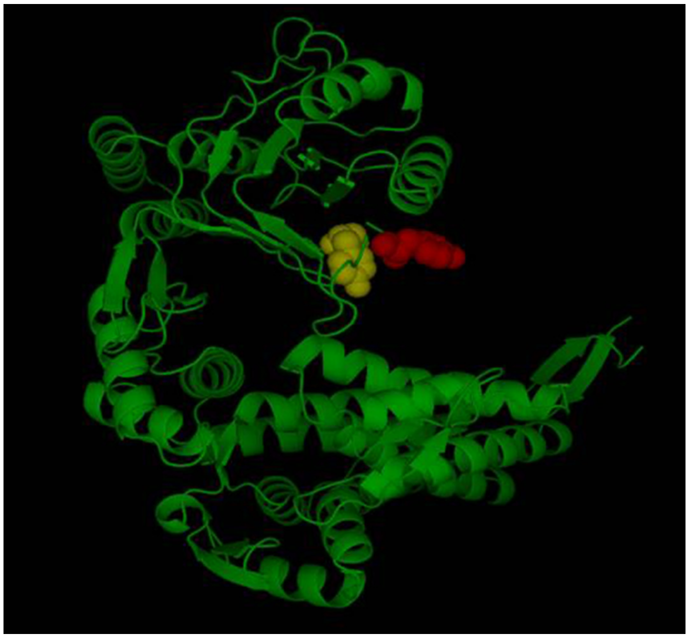

Supplementary Figure S1: Three-dimensional model of HSP60 monomer. Three-dimensional model of HSP60 monomer showing the positions Y222 (yellow) and Y226 (red) in the apical domain. This domain is crucial for co-chaperonin (HSP10) and substrate binding by HSP60.
